# Trans-interlamina percutaneous endoscopic cervical discectomy for symptomatic cervical spondylotic radiculopathy using the new Delta system

**DOI:** 10.1038/s41598-020-67381-z

**Published:** 2020-06-24

**Authors:** Ma Haijun, Zhao Xiaobing, Geng Bin, He Jinwen, Zhao Dacheng, Wang Shenghong, Zhou Honggang, Xia Yayi

**Affiliations:** 1 Department of Orthopaedics, Lanzhou University Second Hospital, No. 82 Cuiying Gate, Chengguan District, Lanzhou, 730030 Gansu People’s Republic of China; 2 Department of Mini-Invasive Spinal Surgery, Third Hospital of Henan Province, No. 198 Funiu Road, Zhongyuan District, Zhengzhou, 450000 Henan People’s Republic of China

**Keywords:** Spinal cord diseases, Peripheral neuropathies, Neurosurgery

## Abstract

To describe the rationale and surgical technique and compare the clinical effect of posterior percutaneous endoscopic cervical discectomy (PPECD) using the Delta system versus that of conventional PPECD (key-hole) surgery for the treatment of symptomatic cervical spondylotic radiculopathy (CSR). A retrospective analysis was performed on 106 single-segment CSR patients between February 2016 and February 2017, 50 of whom underwent conventional PPECD (key-hole), and 56 underwent PPECD using the Delta system. The operative time, intraoperative blood loss, intraoperative complications and postoperative hospital stay were recorded, and the clinical effect was evaluated by the indicators of the Neck Disability Index (NDI), arm-visual analog scale (arm-VAS), neck-VAS, EQ-5D and MacNab classification at the last follow-up. All patients underwent the operation successfully, and 106 patients were followed up. The operative time of the Delta group was 60.47 ± 0.71 min, while the operative time of the key-hole group was 75.46 ± 0.41 min. The difference between the two groups was statistically significant (P < 0.05). However, there was no significant difference between the two groups in terms of blood loss and hospital stay (P > 0.05). The VAS, NDI and EQ-5D scores of the neck and upper limbs in the two groups were significantly better than those before surgery at 1 week after surgery and at the last follow-up (P < 0.05). However, there was no significant difference between the two groups at the last follow-up (P > 0.05). At the last follow-up, there was no significant difference between the two surgical methods when evaluated using the modified MacNab criteria. The imaging results showed that the herniated disc was removed completely and the nerve root was decompressed. The complication rate in the Delta group (3/56, 5.35%) was significantly lower than that in the conventional key-hole group (5/50, 10.0%). PPECD using the Delta system for CSR may be a feasible and promising alternative surgical plan. Compared with the traditional key-hole method, this surgical system can not only provide the surgeon with a larger surgical field of vision but also reduces the operation time and complication rates.

## Introduction

Most cervical spondylotic radiculopathy (CSR) can be alleviated with conservative treatment, but a small number of patients still require surgery for decompression^1^. Traditional anterior cervical decompression and fusion (ACDF) characterized by a sufficient decompression and fusion rate is recognized as the gold standard for the treatment of CSR^1^; however, with the long-term follow-up of ACDF reported in previous studies, the degeneration of adjacent segments, graft subsidence, and loss of intervertebral height, loss of spinal motion in the treated segment, and surgery-related complications have been reported^2,3^.

With the development of minimally invasive techniques in the spine, spinal endoscopy has achieved a clinical effect comparable to that of traditional open surgery and has more obvious advantages in terms of surgical trauma, blood loss, postoperative recovery, length of stay and cost^4–6^. In 2007, PPECD was first reported by Ruetten et al. in Germany and is a cervical posterior "key-hole" technique^7^. Compared with the traditional open-posterior key-hole surgery reported by Frykholm, R^8^, PPECD avoids extensive dissection of the posterior cervical soft tissue, maximizes the bone structure of the spine, and reduces the risk of postoperative segmental instability and axial neck pain.

However, during the actual operation, the diameter of the key-hole operating cannula was approximately 7.5 mm, which easily entered the spinal canal and caused compression of the nerves in the spinal canal. Therefore, we adopted a wider and threaded working channel, namely, the new Delta surgical system, which not only provides the surgeon with a larger surgical field to find and identify bony markers under endoscopy but also facilitates the removal of the lamina by a high-speed burr, thus reducing the operation time. In addition, the threaded channel can increase the friction between the external surface of the instrument and the soft tissue, avoid stimulation of the nerve root and dural sac caused by the left-to-right shaking and sinking of the channel, and improve the safety of the operation. In this study, 56 CSR patients who underwent PPECD using the Delta system were selected, and the report is as follows.

## Materials and methods

### Patients

A retrospective analysis was conducted from February 2016 to February 2017 and included 106 patients in our hospital who had received PPECD treatment for single-segment and unilateral soft cervical disc herniation with limb radiation pain and numbness. Fifty patients received conventional PPECD treatment (group A), and 56 patients underwent PPECD using the Delta instrument (group B). The demographic characteristics of the patients are recorded in Table 1. All operations were performed by the same surgeon.

**Table 1:** Comparison of the baseline data between the key-hole group and Delta group.

Item	The key-hole group (n = 50)	Delta group (n = 56)	P-value
Sex (M/F, n)	23/27	33/23	0.256
Age (mean ± SD, year)	61.19 ± 2.35	61.23 ± 2.56	0.899
Period (mean ± SD, month)	9.12 ± 1.32	9.24 ± 0.18	0.472
Segment (n)			–
C4–5	18	19	–
C5–6	24	25	–
C6–7	8	12	–
Operation time (mean ± SD, min)	75.46 ± 0.41	60.47 ± 0.71	0.00
Estimated blood loss (mean ± SD, ml)	20.33 ± 0.56	20.47 ± 0.34	0.008
Hospital stay (mean ± SD, day)	4.66 ± 0.82	4.45 ± 1.22	0.307
Follow-up period (mean ± SD, month)	24.36 ± 0.89	24.43 ± 0.21	0.569

*SD* standard deviation.

#### Ethics approval and consent to participate

We have conformed that our research was approved by the ethics committee of Third Hospital of Henan Province, all methods were carried out in accordance with relevant guidelines and regulations. Informed consent was obtained from all participants.

**Figure 1:**
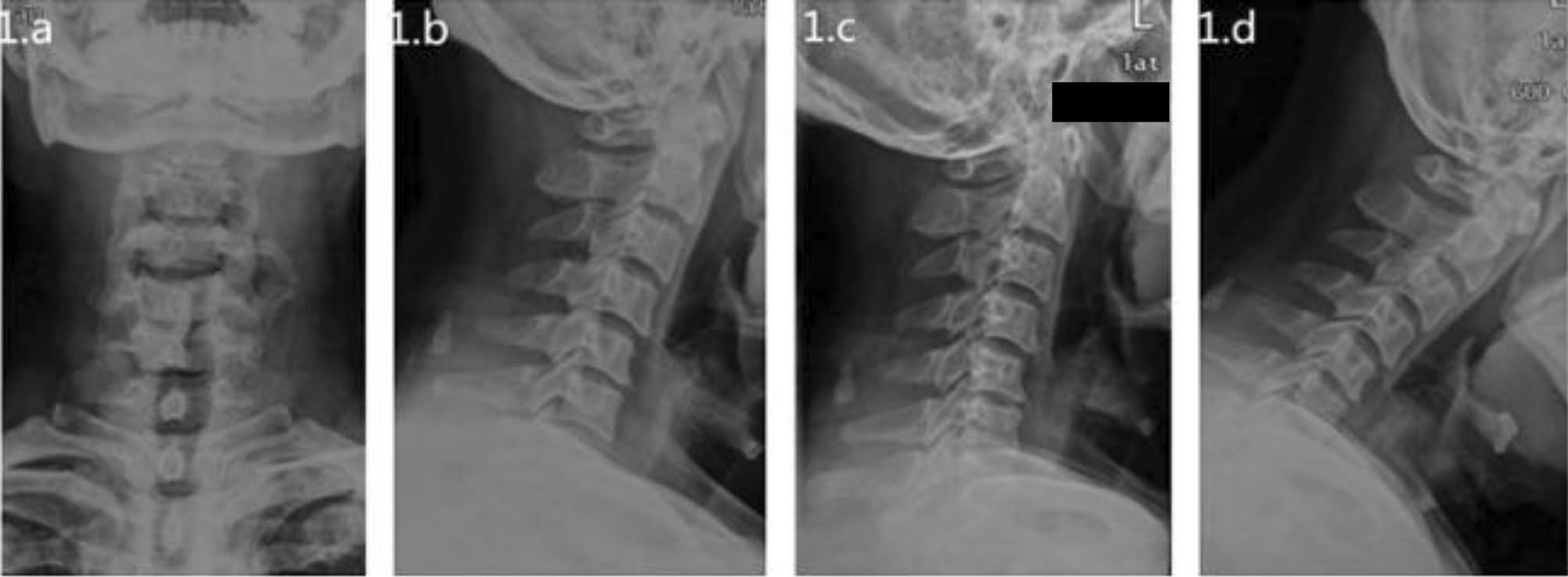
Preoperative cervical X-ray. The anteroposterior and lateral images of the cervical vertebra (**a**) and (**b**) showed degeneration of the cervical vertebra, and the physiological curvature was straightened. Cervical vertebral hyperextension and flexion images (**c** and **d**) showed no cervical vertebral stability.

**Figure 2:**
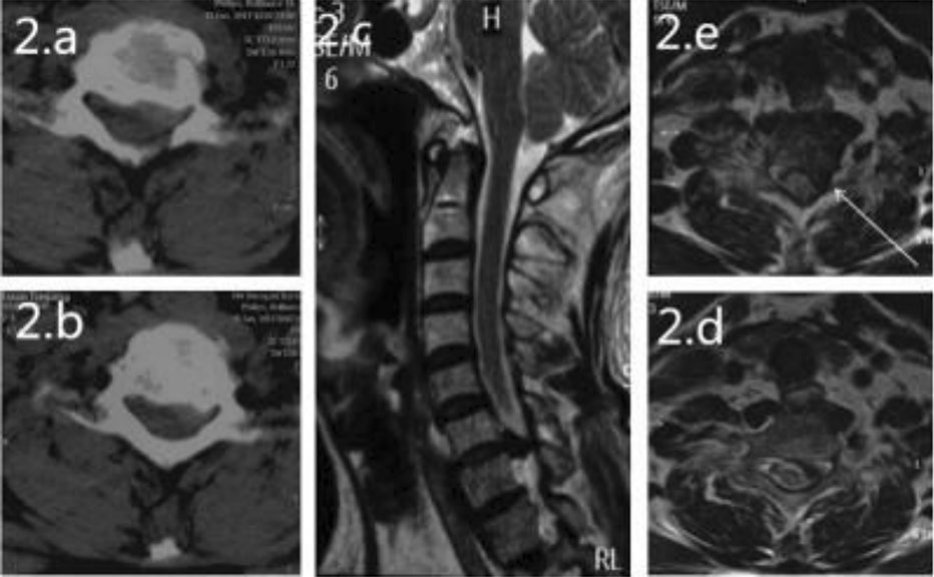
Preoperative MRI and CT. Axial CT (**a**, **b**) demonstrates disc herniation pressing on the left nerve root. Sagittal MRI of the cervical spine (**c**) demonstrates massive disc herniation with nerve root compression at the C6/7 segment. Axial MRI (**e**, **d**) showed a herniated disc compressing on the left nerve root (indicated by the white arrow).

The inclusion criteria of this study were as follows: (1) clinical manifestations of unilateral cervical radiculopathy with arm pain and/or sensory impairment or loss of motor function, without spinal cord compression; (2) imaging suggesting that the disc herniation was located on the lateral side of the spinal cord without cervical instability; and (3) conservative treatment was ineffective for at least 3 months, severe pain or nerve root symptoms can appropriately reduce the time of conservative treatment (Figs. 1, 2).

The exclusion criteria were as follows: (1) cervical surgery history; (2) cervical infection or tumor; (3) multisegment cervical disc herniation with severe degeneration; (4) cervical deformity; (5) severe cervical spinal stenosis, ossification of the posterior longitudinal ligament, or severe disc calcification; and (6) a herniated disc located on the medial or anterior side of the spinal cord.

### Surgical procedures

#### The key-hole group

The patients were placed in a prone position with a chest cushion, the neck slightly flexed and the cranium fixed to stabilize the skull; the bilateral shoulder joints and back skin were pulled down and fixed. We administered general anesthesia through intubation because of the difficulties of airway management in the prone position.

**Figure 3:**
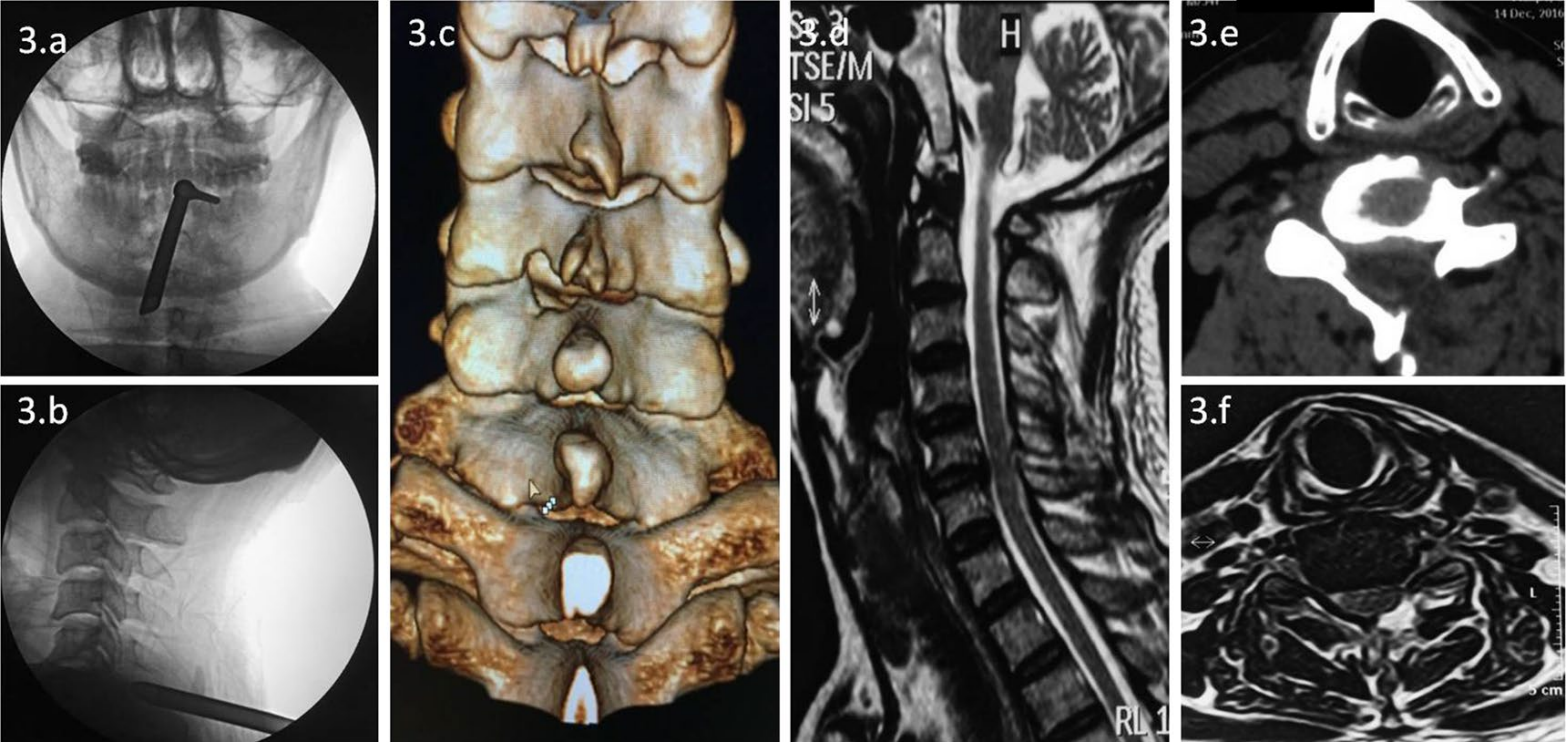
Intraoperative fluoroscopy and postoperative images of the key-hole technique. Intraoperative fluoroscopy confirmed that the working channel was located in the lesion segment (**a**, **b**). The 3D reconstruction image (**c**) shows the bone structure removed by the key-hole technique. Sagittal and axial MRI (**d**, **f**) showed no herniation of the spinal cord or nerve. Axial CT (**e**) showed no disc herniation compressing the spinal cord or nerves.

A surgical incision of approximately 7 mm was made in the lesion segment 1.5 cm from the spinal process on the affected side. Under C-arm fluoroscopy, the surgeon inserted an 18-gauge needle into the lower and medial margins of the inferior articular process of the upper vertebra through the skin incision. Then, the guide wire was inserted, and dilators were inserted along the guide wire and expanded step by step under endoscopy in the working channel. Then, an endoscopic working channel with an inclined surface was inserted along the dilators. The dilators and guide wire were removed and inserted into the spinal endoscope (Fig. 3a, b).

Under spinal endoscopy, soft tissues remaining on the surface of the lamina and facet joint were cleaned with bipolar radiofrequency and nucleus forceps to expose the lower edge of the lamina of the inferior articular process of the upper vertebra in the affected segment, i.e., the "V-point" of exposure. Marked by the V-point, part of the articular processes and lamina were removed under endoscopy with a high-speed burr or Endo-Kerrison punch. To reduce the occurrence of postoperative cervical spine instability, we suggest removing less than 50% of the articular process of the joint range^9^. To ensure that the drilling process did not damage the nerve, our technique was to simply remove the lateral cortical bone and cancellous bone while retaining the medial cortical bone. Pliers were then used to remove the bone cortex and the ligamentum flavum. Then, the epidural venous plexus was coagulated in advance using a bipolar radiofrequency coagulator to maintain a clear visual field. After careful identification of the nerve roots and removal of protruding nucleus pulposus tissue, the operation should be performed gently, avoiding dural extrusion and excessive nerve root pulling. When the dural sac and nerve root appear to fluctuate, decompression is adequate, and the operation can be completed. The instruments were removed, and the incision was closed. After the operation, anti-inflammation and nutritional nerve drugs were applied. On the second day after the operation, the patient could walk and sit.

#### Delta group

**Figure 4:**
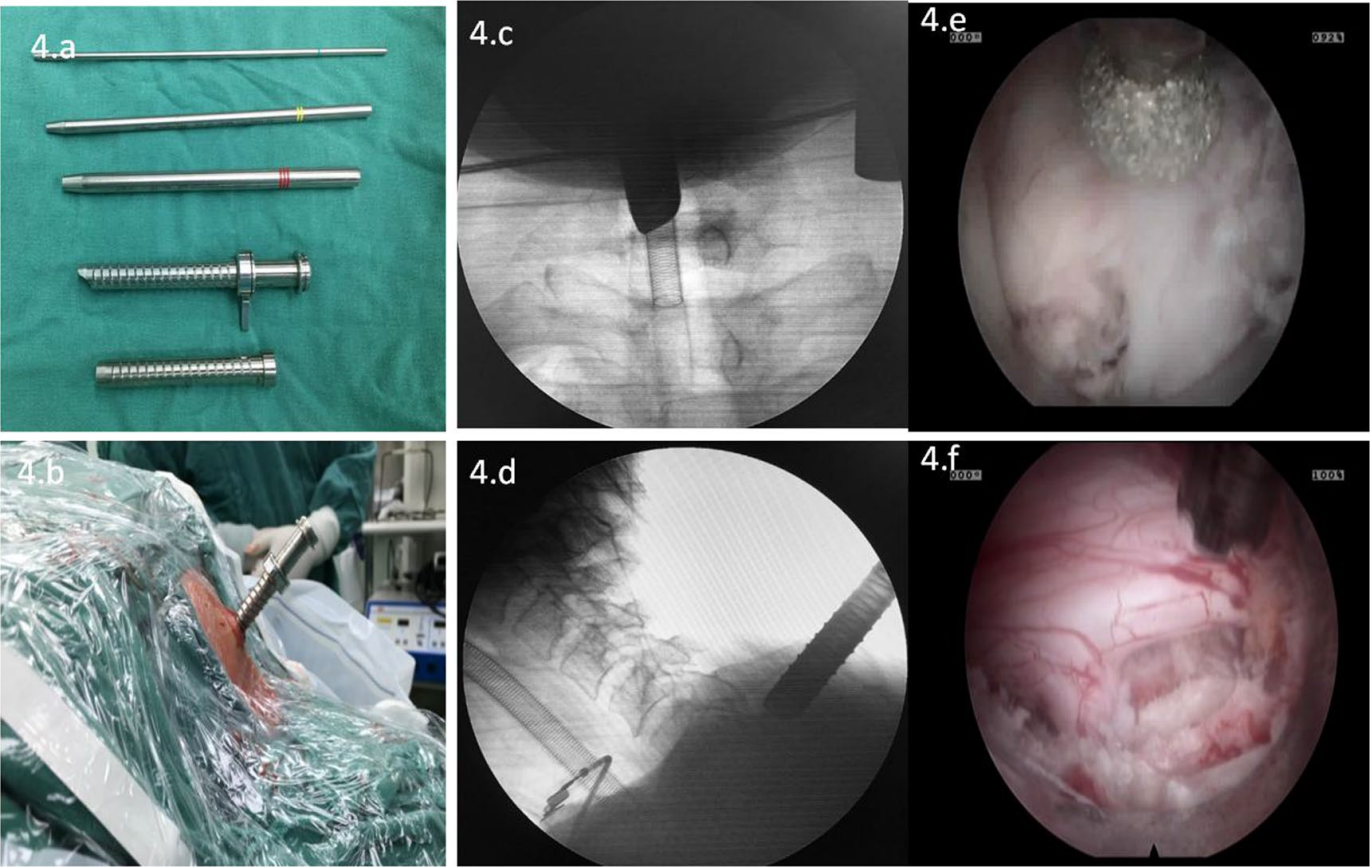
Intraoperative picture and endoscopic images. Delta instrument (**a**). Delta instrument inserted on the lesion segment (**b**). Intraoperative fluoroscopy confirmed that the working channel was located in the lesion segment (**c**, **d**). Endoscopic high-speed burr and drilling to remove part of the lamina and articular process (**e**). Endoscopic visual of the exposed dural sac and nerve root (**f**).

Compared with those in the key-hole group, the operative position and the method of anesthesia were the same as before. A surgical incision of approximately 10 mm was made 1.5 cm beside the affected lateral spinous process in the lesion segment (Fig. 4a–d). Under C-arm fluoroscopy, a 2.0 mm Kirschner wire was vertically anchored through the skin incision to the lateral mass, and the target point at the medial edge of the pedicle projection point on the body surface was determined by fluoroscopy imaging. Step-by-step expansion along the Kirschner wire was performed, and the Delta system was inserted into the working channel. The dilator and Kirschner needle were removed, and the spinal endoscope was inserted.

Under spinal endoscopy, bipolar radiofrequency electrocoagulation and nucleus pulposus forceps were used to clean the remaining soft tissue on the surface of the lamina and facet joints to expose the inner edge of the inferior facet of the upper vertebral body in the lesion segment, the "V point"^10^ and the anchored notch of the Kirchner wire. According to these bony markers, we used a high-speed drill or Endo-Kerrison punch under endoscopy to remove part of the articular process and lamina (Fig. 4e, f). The last decompression procedure and postoperative management were the same as those in the key-hole group.

### Efficacy evaluation

The operative time, intraoperative complications and length of stay were recorded. A visual analog scale (VAS) was used to assess pain, with a score of 0 indicating painlessness and a score of 10 for bipolar the most intense pain that is unbearable. The Neck Disability Index (NDI) was used to assess cervical function with the total score ranging from 0 (no paralysis) to 50 (complete paralysis). The higher the score was, the more serious the disability. The European Quality of Life-5 Dimensions (EQ-5D) were used to evaluate quality of life. At the last follow-up, a modified Macnab standard was used to evaluate the clinical outcomes. Three-dimensional CT and MRI of the cervical vertebra were reexamined after the operation to evaluate the discectomy, nerve decompression and extent of articular process abrasion.

### Statistical analysis

SPSS 22.0 software was used for statistical analysis. The measurement data with a normal distribution are expressed as the mean ± standard deviation. The analysis of variance of repeated measures was used for intragroup comparisons. Two independent sample t-tests were used to compare the measurement data conforming to a normal distribution with homogeneity of variance between two groups, and P < 0.05 was considered statistically significant.

## Results

### Clinical data of the patients

All patients were successfully operated on by Ma HJ and were followed up. In terms of blood loss and length of stay, the difference between the two groups were not statistically significant (P > 0.05). However, the average operation time of Delta group shorter less than that of the key-hole group, and the difference was statistically significant (P < 0.05), as shown in Table 1.

### Clinical results

The VAS scores and NDI scores of neck and upper limb pain in the two groups were significantly improved at 1 week after surgery and at the last follow-up, with statistically significant differences (P < 0.05). After the operation, all the indicators improved significantly with the passage of time. However, there was no significant difference between the two groups at the last follow-up (P > 0.05) as shown in Tables 2, 3.

**Table 2:** Changes in the NDI, arm-VAS values at baseline and each time point postoperatively between the two groups.

Time	NDI score		Arm—VAS score	
	Key-hole group (n = 50)	Delta group (n = 56)	Key-hole group (n = 50)	Delta group (n = 56)
Pre-op	45.67 ± 1.20	47.65 ± 1.35	8.70 ± 0.13	8.45 ± 1.46
Post-op 1 w	22.34 ± 0.81	21.52 ± 1.64	4.10 ± 0.29	3.22 ± 0.54
Last F-up	10.93 ± 0.11	9.14 ± 1.35	1.88 ± 0.26	1.31 ± 0.14
P-value	< 0.01	< 0.01	< 0.01	< 0.01

*VAS* visual analog scale, *NDI* Neck Disability Index.

**Table 3:** Changes in the neck-VAS, EQ-5D values at baseline and each time point postoperatively between the two groups.

Time	Neck—VAS score		EQ-5D score	
	Key-hole group (n = 50)	Delta group (n = 56)	Key-hole group (n = 50)	Delta group (n = 56)
Pre-op	6.75 ± 1.38	6.64 ± 1.28	0.36 ± 0. 6	0.35 ± 0.04
Post-op 1 w	4.19 ± 0.45	3.42 ± 0.44	0.51 ± 0.09	0.57 ± 0.02
Last F-up	1.32 ± 0.45	1.43 ± 0.13	0.71 ± 0.17	0.75 ± 0.13
P-value	< 0.01	< 0.01	< 0.01	< 0.01

*EQ-5D* European Quality of Life-5 Dimensions, Compared with the preoperative value, P < 0.05.

One year after the operation in the Delta group, the clinical efficacy was evaluated by the MacNab standard; there were 40 cases of excellent efficacy, 14 cases of good efficacy, 1 case of fair efficacy, and 1 case of poor efficacy. The excellent and good rate was 96.4%. In the key-hole group, 35 cases were excellent, 12 cases were good, 2 cases were acceptable, and 1 case was poor, with an excellent and good rate of 94.0%. At the last follow-up, there was no significant difference between the two surgical methods when evaluated using the modified MacNab criteria.

### Imaging results

**Figure 5:**
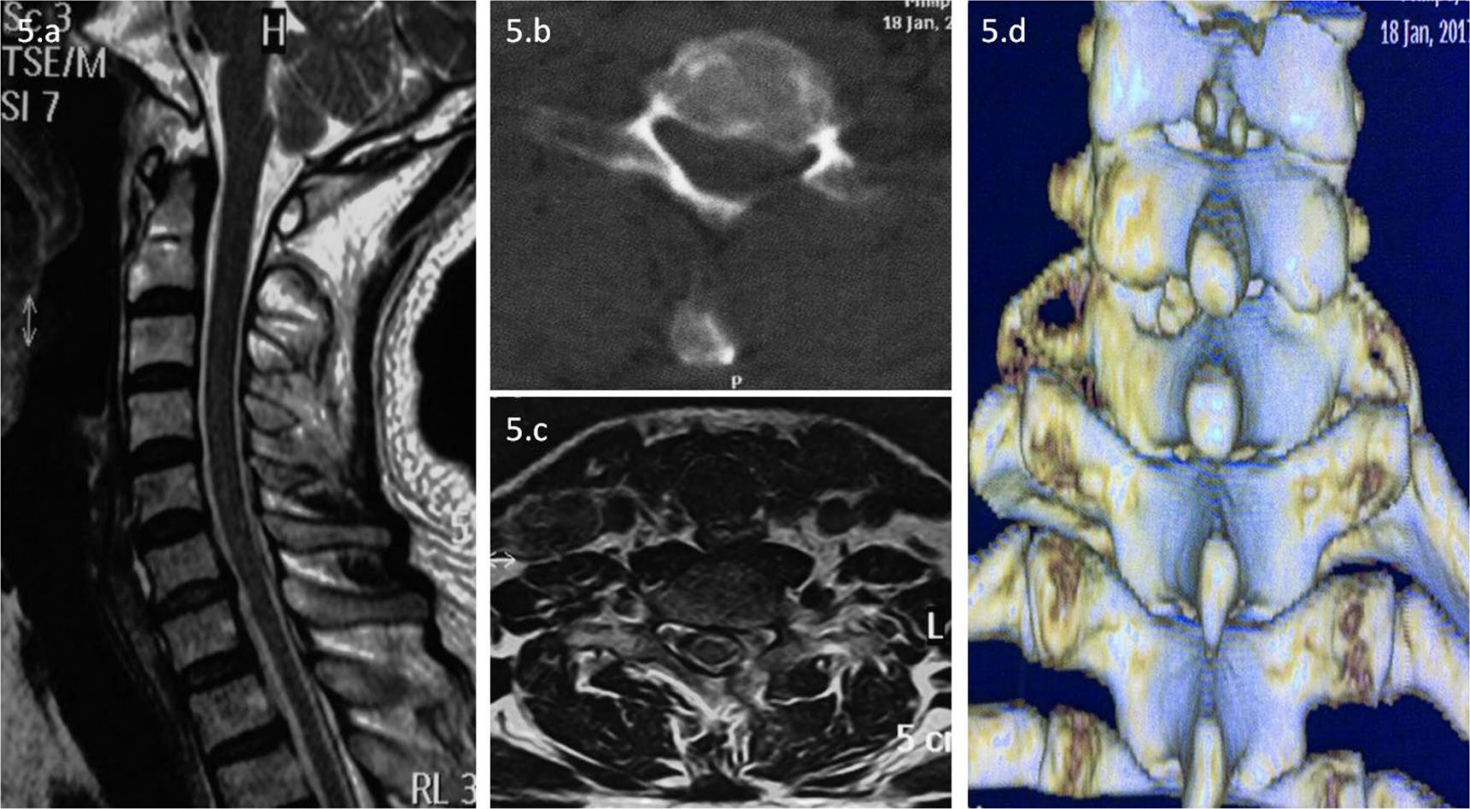
Postoperative MRI and CT. Sagittal and axial MRI (**a**, **c**) showed no disc herniation pressing on the spinal cord or nerves. Axial CT (**b**) showed no disc herniation impairing the spinal cord or nerves. The 3D reconstruction image (**d**) shows the bone structure removed by the Delta technique.

One or two days after surgery, the CT and magnetic resonance imaging (MRI) findings showed that the spinal cord was not compressed, and the herniated disc at C6/7 was resected compared with that on the preoperative images (Figs. 3c–f, 5).

### Complications

A total of eight patients developed postoperative symptoms, including five patients in the key-hole group and three patients in the Delta group.

There were five complications in the key-hole group, including one case of aggravated pain and numbness, two cases of dural tear, one case of decreased muscle strength, and one case of disc herniation recurrence.

Three patients in the Delta group had complications, including one with increased pain, one with numbness, and one with recurrent disc herniation. The complication rate in the Delta group (3/56, 5.35%) was significantly lower than that in the conventional key-hole group (5/50, 10.0%). During the follow-up period, no serious complications, such as death, irreversible nerve injury or even paralysis, occurred in the two groups.

## Discussion

Posterior percutaneous endoscopic cervical discectomy (PPECD) using the Delta system for cervical spondylotic radiculopathy (CSR) may be a feasible and promising alternative surgical plan. This method has the advantages of minimal trauma, minimal blood loss, minimal damage to the soft tissue, minimal disruption to the cervical motion units, fast recovery, no effect on the internal plants and reduced cost. Compared with the traditional key-hole method^7,11^, this surgical system not only provides the surgeon with a larger surgical field of view, but the threaded channel can also increase the friction between the external surface of the instrument and the soft tissue, avoiding the stimulation of the nerve root and dural sac caused by the left-to-right shaking and sinking of the channel. These factors are important because they could reduce the operation time and the rates of complications.

These two kinds of operations showed the most significant differences in operation time and complications. We adopted a wider diameter working channel, known as the new Delta surgical system, which not only provides the surgeon with a larger surgical field to find and identify bony landmarks under endoscopy but also facilitates removal of the lamina by high-speed drilling. In addition, special markers can be created on the lateral mass by using the Kirschner wire anchor, which is convenient in the search of osseous markers, thus reducing the operation time.

The traditional key-hole working channel easily enters the spinal canal and causes compression of the nerves in the spinal canal. Therefore, we adopted a wider and threaded working channel to avoid entering the spinal canal. Moreover, the threaded channel can increase the friction between the external surface of the instrument and the soft tissue, avoid stimulation of the nerve root and dural sac caused by the left-to-right shaking and sinking of the channel, improve the safety of the surgery, and reduce the occurrence of surgical complications, such as postoperative upper limb pain, increased numbness, and even weakened muscle strength.

The VAS scores and NDI scores of neck and upper limb pain in the two groups were significantly improved at 1 week after surgery and at the last follow-up, with statistically significant differences (P < 0.05). After the operation, all the indicators improved significantly with the passage of time. However, there was no significant difference between the two groups at the last follow-up. An evaluation based on the modified MacNab grading criteria showed no significant difference between the two surgical methods. This shows that PPECD using the Delta system may be an effective method to treat CSR.

Recently, with the continuous development of spinal endoscopy systems, endoscopic spinal surgery has been characterized by minimal trauma, rapid recovery of non-fusion, and the retention of movement function^12–15^. In 2007, Ruetten first reported 87 patients with unilateral cervical spondylotic radiculopathy treated by percutaneous posterior endoscopic cervical disc resection (PPECD)^7^. The patients were followed for 2 years. The results showed that 87.4% no longer had arm pain, and 9.2% had only occasional pain. The decompression results were equal to those of conventional procedures. The operation-related trauma was reduced. The recurrence rate was 3.4%. No serious surgical complications occurred. Kim^16^ reported that arm pain improved by more than 90% in 2 patients by 1 week after surgery and in the other patient by 2 months after surgery (excellent outcome according to Macnab's criteria). None of the operations caused instability. Our team retrospectively analyzed 56 cases of unilateral radiculopathy after PPECD using the Delta system, and the results showed that the postoperative pain symptoms in the neck and upper limbs were significantly relieved, and the VAS and NDI scores significantly improved.

Regarding complications, in the key-hole group, pain and numbness were aggravated in one case, and there were two cases of dural tear, one case of decreased muscle strength, and one case of disc herniation recurrence. Three patients in the Delta group had complications, including one with increased pain, one with numbness, and one with disc herniation recurrence. The complication rate in the Delta group (3/56, 5.35%) was significantly lower than that in the conventional key-hole group (5/50, 10.0%). We hypothesized that the lower complication rate in the Delta group was due to the larger endoscopic field of view, which made it easier to distinguish the tissues and reduced stimulation of the nerves in the spinal canal through the working channel. Two patients with disc herniation were treated with endoscopic decompression again. Regarding the aggravated pain, the pain symptoms were significantly relieved after 1 week of treatment with anti-inflammatory and analgesic medicine. After the operation, the patients with temporary sensory disorders of the lower limbs and decreased muscle strength were given nutritional nerve drugs and rehabilitation therapy for 2 weeks to relieve their symptoms. For cerebrospinal fluid leakage, drainage tubes were placed postoperatively and pulled out when the daily drainage volume was less than 20 ml. No low cranial pressure was observed, and the incisions all healed well.

The main disadvantage of our study was the limited number of cases. Despite the good results, it was very difficult to assess the safety and efficacy of this approach in this study. Long-term follow-up and clinical data collection will be required in the future.
